# Molecular epidemiology and antibiotic resistance of staphylococci other than *Staphylococcus aureus* in children in Cape Town, South Africa

**DOI:** 10.3389/fmicb.2023.1239666

**Published:** 2023-08-02

**Authors:** Remous Ocloo, Mae Newton-Foot, Wilma Ziebuhr, Andrew Christopher Whitelaw

**Affiliations:** ^1^Division of Medical Microbiology and Immunology, Department of Pathology, Stellenbosch University, Stellenbosch, South Africa; ^2^National Health Laboratory Service, Tygerberg Hospital, Cape Town, South Africa; ^3^Institute of Molecular Infection Biology, University of Wuerzburg, Wuerzburg, Germany

**Keywords:** *Staphylococcus* other than *S. aureus*, non-aureus staphylococci, *Staphylococcus borealis*, South Africa, Africa, antibiotic resistance

## Abstract

**Introduction:**

Staphylococci other than *Staphylococcus aureus* (SOSA) have emerged as significant pathogens in healthcare settings, particularly among patients with indwelling devices and immunocompromised individuals. *Staphylococcus epidermidis, Staphylococcus haemolyticus* and *Staphylococcus hominis* are the most common commensal SOSA species and are implicated in infections such as endocarditis and bacteremia. SOSA infections in neonates and children have been reported globally. Recent increases in antibiotic resistance and virulence among SOSA strains in clinical settings have highlighted the need to describe the reservoirs of SOSA to enable monitoring of these emerging pathogens.

**Methods:**

Stool samples were collected from 150 healthy children from Cape Town communities between 2017 and 2020. Staphylococci were isolated, identified using mass-spectrometry, and antimicrobial susceptibility testing and Illumina whole genome sequencing were performed.

**Results:**

Among the participants, 50 (33.3%) were colonized by SOSA, with *S. haemolyticus* (*n* = 38; 25.3%) being the most common, followed by *S. hominis* (*n* = 5; 3.3%) and *Mammalicoccus sciuri* (*n* = 5; 3.3%). Out of the 77 initially isolated *S. haemolyticus* strains, 23 were identified as *Staphylococcus borealis* through whole genome sequencing. All *S. haemolyticus* isolates (*n* = 49) were methicillin resistant, with 65.3% (*n* = 32) harbouring *mecA*. In *S. haemolyticus*, SCC*mec* type VIII(4A) was detected in 42.0% of ST9 isolates while non-*mecA* methicillin resistant *S. haemolyticus* isolates were mostly ST49 (41.1%). Additionally, 16 (50.0%) *S. haemolyticus* strains contained non-typeable SCC*mec* elements.

**Discussion:**

High rates of methicillin resistance were identified among colonizing SOSA in Cape Town, increasing the risk of transmission to clinical settings. This study also identified a new species, *S. borealis*, for the first time in Africa.

## Introduction

*Staphylococcus* species are a major component of the human skin and mucosa microbiota. Also, fecal carriage of staphylococci is widespread in humans, although the number of bacteria detectable in stool samples is usually low (Akinkunmi and Lamikanra, 2012). Commensal staphylococci can become pathogenic when there is a break in the skin or mucosal barrier as well as during the insertion of indwelling medical devices (Kateete et al., 2020). Staphylococci were previously categorized as coagulase-negative or coagulase-positive based on their ability to produce the coagulase enzyme (Gómez-Sanz et al., 2019), and the term coagulase-negative staphylococci became widely used to encompass all staphylococci other than the well-recognized human pathogen *Staphylococcus aureus*. However, due to the coagulase-variable nature of some species, the term staphylococci other than *Staphylococcus aureus* (SOSA) has been introduced to better differentiate *S. aureus* and other (“non-aureus”) staphylococci (Humphries et al., 2021).

Neonates may be colonized with SOSA through breastfeeding, and high rates of colonization ranging from 92.9%–98.9% have been reported (Soeorg et al., 2017).

Antibiotic resistance (ABR) is a worldwide and common problem in staphylococci, including SOSA. The prevalence of colonization with methicillin-resistant SOSA (MR-SOSA) ranges widely from 30.2% to as high as 89.3% in healthy individuals in Nigeria (Akinkunmi and Lamikanra, 2010) and Brazil (Ternes et al., 2013). SOSA are often considered a reservoir for ABR and virulence genes, which can be transferred to other pathogenic bacteria such as *S. aureus* (Nemeghaire et al., 2014). Colonization by resistant SOSA could therefore facilitate the spread of ABR genes into other species and contribute to the general infection burden by antibiotic resistant bacteria (Jamaluddin et al., 2008). The staphylococcal cassette chromosome *mec* (SCC*mec*) are mobile genetic elements which usually carry *mec* genes (i.e., *mecA, mecB, mecC*) that encode alternative penicillin-binding proteins, mediating resistance to nearly all β-lactam antibiotics (Saber et al., 2017). SCC*mec* elements have their evolutionary origin most likely in *Mammaliicoccus sciuri* (previously known as *Staphylococcus sciuri*) and have been transferred to *S. aureus* and other SOSA species. Fourteen SCC*mec* types (I–XIV) have been described in *S. aureus* (Urushibara et al., 2019), of which SCC*mec* types I–IX have been reported in SOSA, mostly in *Staphylococcus epidermidis*, *Staphylococcus haemolyticus,* and *Staphylococcus hominis*. However, the majority of SCC*mec* elements in SOSA are non-typeable using available methods, due to the high recombination rates in SOSA (Miragaia, 2018), which increases their diversity.

Apart from *S. epidermidis* for which surveillance data are well established (Thomas et al., 2007), there is limited multi-locus sequence typing (MLST) information and molecular epidemiology data available for SOSA. Nevertheless, *S. haemolyticus* ST239 has been associated with SCC*mec* type III (Lin et al., 2022; Montelongo et al., 2022) and *S. hominis* ST16 and ST23 have been identified in outbreaks in Spain (Zhang et al., 2013). These STs were reported in pathogenic SOSA, but there is a lack of information on the distribution of STs in commensal SOSA in Africa and in Sub-Saharan Africa. With this study we would like to fill this knowledge gap and analyse the gastrointestinal carriage and population structure of ABR SOSA isolates in healthy children in Cape Town communities.

## Methods

### Study population

This sub-study forms part of a parallel-group, two-arm, cluster-randomized, and double-blind placebo-controlled multidrug resistant (MDR) tuberculosis (TB) prevention trial (TB-CHAMP-http://www.isrctn.com/ISRCTN92634082), conducted by the Desmond Tutu TB Centre (DTTC), Stellenbosch University. TB-CHAMP is evaluating the efficacy of levofloxacin prophylaxis for MDR-TB. Healthy children aged <5 years with a household contact with confirmed MDR-TB were enrolled and randomized to 6 months of either levofloxacin or placebo treatment in a 1:1 ratio. Preceding the initiation of treatment with levofloxacin or placebo, baseline stool samples were collected from the enrolled children. Stool samples were collected as per DTTC stool collection SOP (Supplementary 1). The stool samples were transported to the Division of Medical Microbiology and Immunology on ice and stored at −80°C. One hundred and fifty (150) baseline stool samples were included in this study.

### Isolation and identification of staphylococci

A loopful of each stool sample was inoculated in 2 mL Lysogeny broth and incubated aerobically for 6 h at 37 °C to promote bacterial growth (Marincola et al., 2021). The broth was then sub-cultured onto 5% sheep blood agar plates supplemented with 10 μg/mL each of nalidixic acid and colistin (Thermofisher, City, United States) to suppress the growth of Gram-negative bacteria. Isolates were selected based on colony morphology consistent with staphylococci. A maximum of five morphologically similar or 10 morphologically dissimilar colonies per sample were randomly selected. This approach enabled us to describe the distribution of different strains colonizing an individual. Staphylococcal isolates were further confirmed using catalase and bile esculin agar (both from NHLS Media Laboratory, Greenpoint, South Africa). Isolates that were bile esculin negative but catalase positive represent staphylococci. The isolates were speciated using matrix-assisted laser desorption/ionization time-of-flight mass spectrometry (MALDI-TOF/MS; VITEK® MS, BioMérieux, France).

### Antibiotic susceptibility testing

Antibiotic susceptibility testing (AST) was performed on *S. aureus* and the most common SOSA species using the Kirby Bauer disc diffusion method, to sub-select isolates for further analysis. The following antibiotic discs (MAST Group, United Kingdom) were used: cefoxitin (30 μg), clindamycin (2 μg), erythromycin (15 μg), rifampicin (5 μg), linezolid (30 μg), and trimethoprim/sulfamethoxazole (1.25/23.75 μg). Susceptibility testing results were interpreted using the Clinical and Laboratory Standards Institute (CLSI) guidelines for 2020 (CLSI, 2020).

Isolates of the same species from the same participant with identical susceptibility patterns to the above antibiotics were presumed to represent the same strain lineage, and only one isolate per participant was included for further molecular analysis. If zone size diameters differed by ≥4 mm (regardless of categorical interpretation) for any antibiotic, isolates were considered potentially unrelated and were both included for further analysis.

The selected isolates were subjected to additional Kirby Bauer disc diffusion and whole genome sequencing. Additional susceptibility testing included: ceftaroline (30 μg), chloramphenicol (30 μg), fusidic acid (10 μg), levofloxacin (5 μg), and tetracycline (30 μg) (all antibiotic discs from MAST Group, United Kingdom). Vancomycin susceptibility testing was performed using vancomycin-supplemented (6 μg/mL) brain heart infusion media (NHLS Media Laboratory).

### DNA extraction

DNA was extracted from the selected isolates using the Quick-DNA^™^ Fungal/Bacterial Miniprep Kit (Zymo Research, United States) as per the manufacturer’s instructions. The concentration and purity of the DNA extracts were assessed using a BioDrop spectrophotometer (BioDrop, United Kingdom), with acceptable A260/A230 and A260/A280 ratios ranging from 1.5–2.4 and 1.7–1.9, respectively. DNA with purity ratios which were out of range were further purified using ethanol precipitation (Nel Van Zyl et al., 2020). The DNA concentrations were confirmed using a Qubit 4 fluorometer (ThermoFisher, United States). The extracted DNA was stored at −20°C.

### Whole genome sequencing

Whole genome sequencing was performed at the Institute of Molecular Infection Biology, University of Wuerzburg, Germany, using Nextera XT DNA Library Prep kits and the NextSeq 2000 (Illumina). Raw sequence reads were automatically generated, checked for quality, trimmed, and *de novo* assembled using FastQC v0.11.7, Trimmomatic v0.39 and SPAdes v3.15.5, respectively. The assembled genomes were submitted to rMLST^1^ for species identification. Genomes with more than one staphylococcal species identified were further investigated using EZBioCloud^2^ for average nucleotide identity (ANI). Identity was assigned based on ≥95% ANI. Prokka v1.14.5 was used for genome annotation of common staphylococcal species. PubMLST^3^ and SCC*mec*Finder^4^ were used to predict sequence types (ST) and SCC*mec* types, respectively. Resistance genes were identified using RGI v6.00.

## Results

### Distribution of SOSA

Fifty-nine of the 150 children (39.3%) were colonized with any *Staphylococcus* spp.: nine (6.0%) were colonized with *S. aureus* only, 37 (24.7%) were colonized by SOSA only, and 13 (8.7%) were co-colonized by *S. aureus* and SOSA. Carriage rates of specific SOSA species include: *S. haemolyticus* (25.3%; *n* = 38), *M. sciuri* (3.3%; *n* = 5), *S. hominis* (3.3%; *n* = 5), *S. epidermidis* (2.0%; *n* = 3), *S. simulans* (1.3%; *n* = 2), *S. saprophyticus* (1.3%; *n* = 2) and *S. capitis* (0.7%; *n* = 1).

In total, 97 *S. aureus* and 238 SOSA isolates were obtained, comprising *S. haemolyticus* (67.7%; *n* = 161), *M. sciuri* (8.8%; *n* = 21), *S. hominis* (7.9%; *n* = 19), *S. epidermidis* (7.9%; *n* = 19), *S. saprophyticus* (3.3%; *n* = 8), *S. simulans* (2.5%; *n* = 6), and *S. capitis* (1.6%; *n* = 4).

### Carriage of antibiotic resistant SOSA

The initial AST screening was performed on 335 staphylococcal isolates (Table 1). All isolates were susceptible to linezolid except five *S. aureus* and almost all the *S. aureus* isolates (92/97, 95%) were methicillin susceptible. The *S. aureus* isolates were not analyzed further.

**Table 1:** Proportion of children colonized by antibiotic resistant staphylococci.

Species	Number of children colonized. *N* = 150 (%)	Total number of isolates. *N* = 335 (%)	FOX (%)	TS (%)	LZD (%)	RP (%)	CD (%)	E (%)
*S. haemolyticus*	38 (25.3)	161 (48.0)	38 (25.3)	18 (12.0)	0	6 (4.0)	10 (6.6)	25 (16.6)
Other SOSA	18 (12.0)	77 (22.9)	14 (9.3)	7 (4.6)	0	2 (1.3)	6 (4.0)	9 (6.0)
Any SOSA	45 (30.0)	238 (71.0)	42 (28.0)	23 (15.3)	0	7 (4.7)	15 (10.0)	29 (19.3)
*S. aureus*	22 (14.6)	97 (28.9)	1 (0.6)	1 (0.6)	1 (0.6)	1 (0.6)	4 (2.6)	10 (6.6)

Oxacillin (FOX), trimethoprim sulfamethoxazole (TS), rifampicin (RP), clindamycin (CD) erythromycin (E), chloramphenicol (C), ceftaroline (CPT), tetracycline (T), levofloxacin (LEV), linezolid (LZD). Other SOSA represents *S. epidermidis*, *S. saprophyticus*, *S. simulans*, *M. sciuri*, *S. hominis*, and *S. capitis*.

From the most common SOSA species colonizing the children, 77 *S. haemolyticus*, six *S. hominis* and five *M*. *sciuri* were sub-selected for further AST and whole genome sequence (WGS) based on their initial AST profile. Isolates were selected to represent all the AST profiles from each participant.

**Figure 1:**
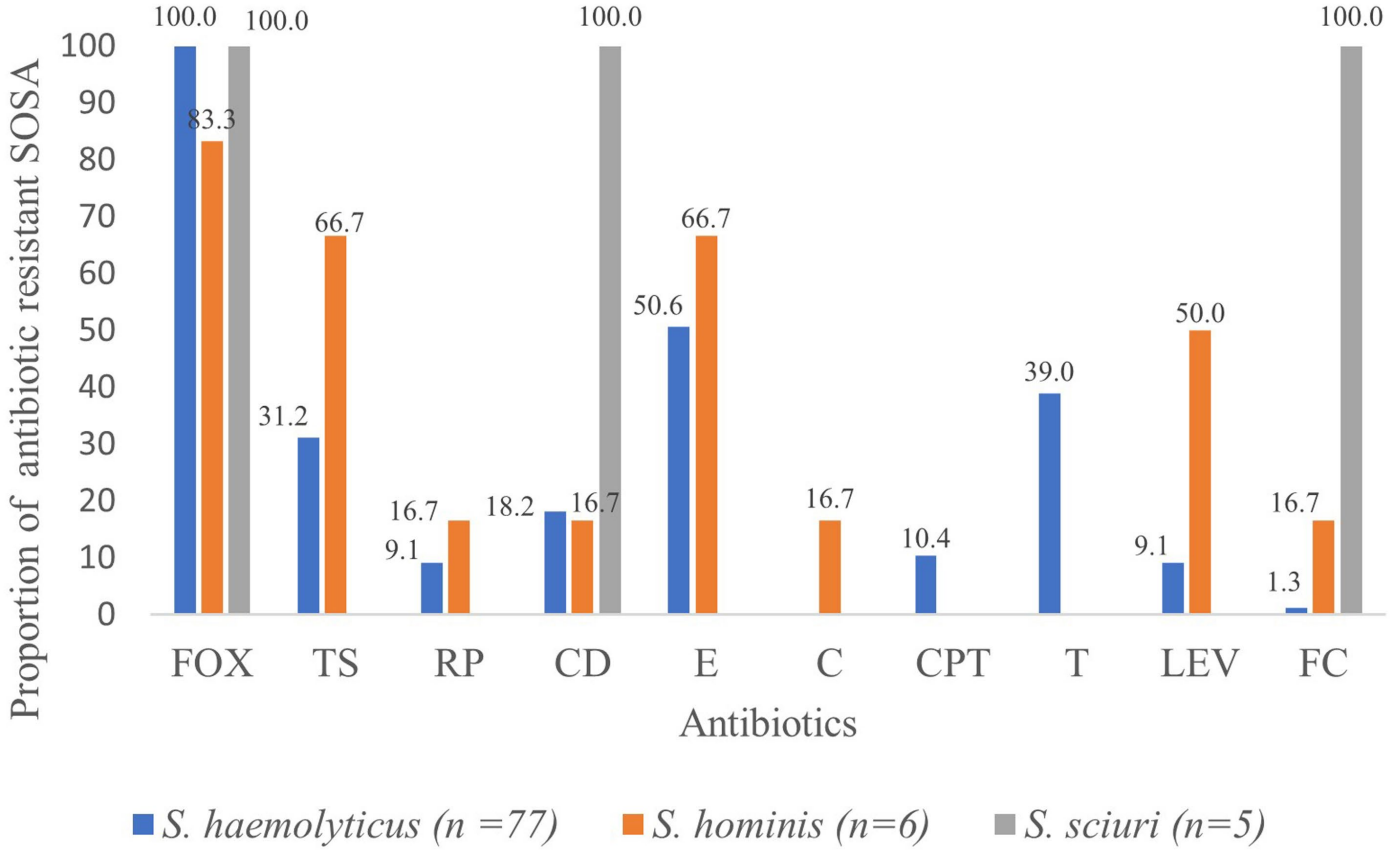
Proportion (%) of antibiotic resistant SOSA. oxacillin (FOX), trimethoprim sulfamethoxazole (TS), rifampicin (RP), clindamycin (CD) erythromycin (E), chloramphenicol (C), ceftaroline (CPT), tetracycline (T), levofloxacin (LEV), fusidic acid (FC).

Methicillin resistance was common, and all *S. haemolyticus* isolates were methicillin resistant. Resistance to tetracycline (*n* = 30; 38.9%) and ceftaroline (*n* = 8; 10.4%) was only seen in *S. haemolyticus,* but these isolates all retained susceptibility to chloramphenicol (Figure 1). All five *M. sciuri* isolates were resistant to clindamycin and fusidic acid. All isolates of all species were susceptible to vancomycin.

High rates of colonization with resistant *S. haemolyticus* were observed for oxacillin (*n* = 38; 25.3%), erythromycin (*n* = 25; 16.6%), tetracycline (*n* = 25; 16.6%) and trimethoprim/sulfamethoxazole (*n* = 18; 12.0%), with lower rates of colonization with resistant strains of the less frequently isolated *S. hominis* and *M. scuiri.* Twenty-eight (18.6%) of the children were colonized by multidrug resistant *S. haemolyticus*; resistant to at least three classes of antibiotics.

### Whole genome sequence analysis of SOSA

#### Species Identification of SOSA

The WGS of five *S. haemolyticus* isolates were contaminated with foreign DNA and excluded from further analysis, leaving 72 analyzable isolates. All six *S. hominis* and five *M. sciuri* were confirmed by WGS analysis. Of the 72 isolates which had been identified as *S. haemolyticus* based on MALDI-TOF, 23 were identified as *S. borealis* through WGS. The average nucleotide identity (ANI) of the 23 isolates were ≥95% when compared to *S. borealis* and ≤91% when compared to *S. haemolyticus.* The antibiotic resistance profiles of *S. borealis* and *S. haemolyticus* were similar. However, the rate of trimethoprim sulfamethoxazole resistance is lower while clindamycin resistance is higher in *S. borealis* (Supplementary 2).

#### Identification of antibiotic resistance genes

The penicillin resistance gene *blaZ* was common in both *S. haemolyticus* (85.7%) and *S. borealis* (86.9%) (Table 2). Three *S. hominis* and two *M. sciuri* also harbored the *blaZ* gene. Supplementary 3 shows ABR genes, SCC*mec* and sequence types identified in *S. hominis* and *M. sciuri*. The methicillin resistance gene *mecA* was more frequent in *S. borealis* (86.9%) than *S. haemolyticus* (56.3%), and tetracycline resistance genes were only detected in *S. haemolyticus* and *S. borealis*. All *S. haemolyticus* isolates harbored the *norC* gene which is associated with fluoroquinolone resistance. Although not tested phenotypically, all isolates harbored the disinfectant resistant gene *sdrM* (Supplementary 3). No resistance genes were identified for ceftaroline.

**Table 2:** Common antibiotic resistance genes, sequence types and SCC*mec* types identified in *S. haemolyticus* and *S. borealis.*

	*S. haemolyticus n* = 49 (%)	*S. borealis n* = 23 (%)
*Antibiotic resistance genes*		
Methicillin		
*mecA*	32 (56.3)	20 (86.9)
Trimethoprim-sulfamethoxazole		
*dfr*G	23 (46.9)	17 (73.9)
*dfr*C	15 (30.6)	0
Tetracycline		
*tet(K)*	2 (4.0)	0
*tet(L)*	3 (4.0)	9 (39.1)
*tet(M)*	11 (22.4)	0
Erythromycin		
*ermA*	4 (8.1)	0
*ermC*	9 (18.3)	10 (43.4)
*mrsA*	35 (71.4)	3 (13.0)
Fluoroquinolone		0
*qacA*	1 (2.0)	12 (52.1)
*qacB*	1 (2.0)	0
*norC*	49 (100.0)	0
*gyrA*	7 (14.2)	0
*Sequence types*		
ST1	2 (4.0)	NA
ST3	1 (2.0)	
ST9	19 (38.7)	
ST29	2 (4.0)	
ST30	5 (10.2)	
ST49	10 (20.4)	
ST128^a^	1 (2.0)	
ST129^a^	1 (2.0)	
ST130^a^	1 (2.0)	
ST131^a^	1 (2.0)	
ST132^a^	1 (2.0)	
ST133^a^	1 (2.0)	
ST135^a^	4 (8.1)	
**SCC*mec* types**	**(*n* = 32)**	**(*n* = 20)**
SCC*mec*_type_I(1B)	1 (3.1)	0
SCC*mec*_type_Vc(5C2&5)	2 (6.2)	0
SCC*mec*_type_V(5C2)	2 (6.2)	0
SCC*mec*_type_VI(4B)	1 (3.1)	4 (17.3)
SCC*mec*_type_VIII(4A)	10 (31.2)	0
Non-typeable	16 (50.0)	16 (80.0)

a Novel sequence types, NA, not applicable—no MLST scheme is available for *S. borealis*.

#### Sequence typing and staphylococcal cassette chromosome *mec* typing of SOSA

ST9 was the most common sequence type amongst *S. haemolyticus* (*n* = 19; 38.7%), followed by ST49 (*n* = 10; 20.4%); and 7 novel *S. haemolyticus* sequence types were identified (Table 2).

Diverse SCC*mec* types were detected, the most common being SCC*mec* type VIII(4A) (*n* = 10; 31.2%). SCC*mec* type VIII(4A) was detected in 8/19 (42%) ST9 isolates while ST49 was detected mostly amongst non-*mecA* methicillin resistant *S. haemolyticus* (*n* = 7/17; 41.1%) isolates. Multiple *S. haemolyticus* were isolated from 14 children based on AST profile, 10 of whom were colonized by at least two different STs.

All *M. sciuri* and *S. hominis* were assigned novel sequence types (4 each, Supplementary 3). All *S. borealis* were non-typeable due to the absence of a typing scheme for this species. SCC*mec* type VI(4B), SCC*mec* type III(3A) and SCC*mec* type VIII(4A) were identified amongst *S. borealis* (*n* = 4)*, M. sciuri* (*n* = 2) and *S. hominis* (*n* = 2) respectively. Sixteen (50.0%) *S. haemolyticus* and 16 (80.0%) *S. borealis* contained non-typeable SCC*mec* elements.

## Discussion

In this study, phenotypic and genotypic methods were used to characterize SOSA isolates. The whole genome sequencing analysis has provided additional information on antibiotic resistance genes, strain types and speciation.

High rates of colonization with SOSA have been reported in healthy children: 37%–42% in Nigeria (Akinkunmi and Lamikanra, 2010), and in Scotland (Jackson et al., 2000). Of the children included in this study, 33.3% were colonized by SOSA. The species of SOSA isolated from this study were similar to those isolated by Domínguez et al. (2002) in Spanish children and Akinkunmi and Lamikanra (2010) in Nigerian children; however those studies also identified *S. warneri* and *S. caprae,* and *S. schleiferi*, *S. lugdunesis* and unspeciated SOSA, respectively. In both studies, *S. epidermidis* was the most common SOSA colonizing children, Nigeria (30.2%) and Spain (38.5%), while in our study *S. haemolyticus* was the most commonly isolated (25.3%). This demonstrates that carriage of SOSA species in the faecal samples of children/humans may differ between geographical locations. Together with *S. capitis*, which was also reported in all three studies, these SOSA have been implicated in nosocomial infections associated with high rates of antibiotic resistance (Decalonne et al., 2020).

Due to the small numbers of the other SOSA isolated in this study, the discussion will focus on *S. haemolyticus*. *S. haemolyticus* and *S. borealis* are discussed together due to the inability of commonly used species identification methods to distinguish them. All *S. haemolyticus* isolates (100.0%) from this study were methicillin resistant. This is higher than the 46.2% (*n* = 12) seen in Nigeria, 50.0% (*n* = 1) in Spain and 10.0% in public settings in London (Domínguez et al., 2002; Akinkunmi and Lamikanra, 2010; Xu et al., 2018). This high methicillin resistance rate threatens empiric treatment practices in Cape Town, South Africa. This may also highlight the need to review the use of antibiotics in Cape Town communities.

The tetracycline resistance rate in *S. haemolyticus* (39.0%) is similar to that described in carriage isolates in Nigeria (30.8%). Although tetracycline is not approved for use in children, the high resistance rates seen in Nigeria and South Africa may reflect the use of this antibiotic in adult populations, as well as food animals and agriculture in Africa (Mshana et al., 2021).

Resistance to erythromycin (50.6%) was similar to what was reported in Nigeria (53.8%). Resistance to trimethoprim-sulfamethoxazole (31.2%) was lower than the 53.8% reported in Nigeria while clindamycin resistance was slightly higher (18.2% vs. 11.5%). The lower rate of resistance of *S. haemolyticus* to trimethoprim-sulfamethoxazole in this study is surprising since trimethoprim-sulfamethoxazole is one of the commonest antibiotics used extensively for prophylaxis in HIV infected individuals in South Africa (Takuva et al., 2019). In this study, all isolates were susceptible to vancomycin, which is similar to what was reported by Domínguez et al. (2002) in Spanish children, however 15.4% vancomycin resistance was reported in Nigeria (Akinkunmi and Lamikanra, 2010).

High rates (30%) of multidrug-resistant SOSA (MDR-SOSA) colonization have also been reported in neonates in Brazil (Ternes et al., 2013), this is higher than the 18.6% observed in this study.

The methicillin resistance gene *mec*A was detected in only 65.3% of *S. haemolyticus* isolates, despite all being methicillin resistant. The *mecA* detection rate was higher than 22.2% reported in non-healthcare settings in the United Kingdom (Xu et al., 2018). Resistance to β-lactams such as oxacillin has been associated with *mec*A and rarely *mec*C. The absence of *mec*A in 34.7% of methicillin-resistant *S. haemolyticus* may be due to challenges in using cefoxitin and oxacillin for screening for methicillin resistance in non*-aureus* staphylococci, as described by Yang et al. (2022) and Humphries et al. (2021). Other novel resistance mechanisms may be involved, such as elevated expression of *blaZ,* which has been shown in some SOSA isolates, although this has not been conclusively linked to methicillin resistance (Black et al., 2011).

This study is the first to identify *S. borealis* in Africa*. S. borealis* shares 861 core genes with *S. haemolyticus,* representing 34.6% of its genome (Pain et al., 2020). *S. borealis* cannot be distinguished from *S. haemolyticus* using the three bacterial identification system commonly used globally (MALDI-TOF, Vitek AES system, and *16S RNA* sequencing) which raises the possibility that *S. borealis* may be wrongly identified as *S. haemolyticus*. However, Pain et al. (2020) described distinctive phenotypic characteristics of *S. borealis* using strains from Norway, which may be useful in distinguishing the species. The high clindamycin resistance in *S. borealis* is concerning and its impact on public health deserves further investigation. We recommend a future study using a large sample size, describing the distribution, antibiotic resistance, and virulence of *S. borealis* in South Africa and globally.

The study reveals the limitations of strain typing in SOSA globally. We identified novel strain types of *S. haemolyticus, S. hominis* and *S. sciuri*, but due to the limited *S. borealis* isolates which have been described, no typing system is available for this species. SCC*mec* types could only be assigned to 42.8% of methicillin resistant isolates, across all species. SCC*mec* type VIII is most common in *S. haemolyticus* in our setting, however, SCC*mec* type VIII has only been reported in clinical *S. hominis* and *S. epidermidis* in Tunisia and Malaysia, respectively (Bouchami et al., 2011; Sani et al., 2014). Majority of SCC*mec* elements from methicillin resistant SOSA (57.1%) were non-typeable; this not surprising due to the high recombination rate in SOSA and partly because the current SCC*mec* typing scheme is dedicated to *S. aureus* and therefore not ideal for SOSA (Ternes et al., 2013; Ikhimiukor et al., 2023). The International Working Group on the Staphylococcal Cassette Chromosome elements^5^ does not annotate SCC*mec* types in SOSA due to the high complexity of the elements found in SOSA. We, therefore, recommend that new approaches be explored to characterize/type SCC*mec* elements in SOSA to better represent the SCC*mec* structures in *Staphylococcus* other than *S. aureus* (SOSA). The use of AST profiles to identify potential strain differences among the isolated staphylococci has many limitations, but it was a pragmatic approach to focus the molecular analysis of isolates with a likelihood of being different. It is encouraging that of the 14 participants who had multiple isolates with different AST profiles, 10 were confirmed to be different STs. However, we cannot comment on the possibility that isolates with the same AST profile from the same participant may have belonged to different STs.

## Conclusion

The findings suggests that children in Cape Town are colonized by multidrug resistant SOSA. This increases the risk of dissemination into clinical settings and poses a risk to empiric treatment practices. The study highlights the need for improving speciation and epidemiological tools such as multilocus sequence typing (MLST) and SCC*mec* typing to better describe the population structure of SOSA which can help in designing effective ABR surveillance and control strategies.

## Data availability statement

The novel sequence types presented in this study can be found in online repositories. The names of the repository/repositories and accession number(s) can be found at: https://www.ncbi.nlm.nih.gov/, PRJNA980213.

## Ethics statement

This study was approved by Health Research Ethics Committee (HREC), Stellenbosch University. Written informed consent to participate in this study was provided by the participants’ legal guardian/next of kin.

## Author contributions

RO contributed to the conceptualisation and design of the study, performed laboratory work, whole genome sequence data analysis and interpretation, and wrote the manuscript. MN-F, AW, and WZ contributed to the conceptualisation and design of the study and provided critical feedback on the manuscript. All authors contributed to the article and approved the submitted version.

## Funding

This study was supported by the German Research Council (DFG) through grant ZI665/3-1 and the German Federal Ministry of Education and Research (BMBF) through grant number 01KI2009E and the Harry Crossley foundation.

## Conflict of interest

The authors declare that the research was conducted in the absence of any commercial or financial relationships that could be construed as a potential conflict of interest.

## Publisher’s note

All claims expressed in this article are solely those of the authors and do not necessarily represent those of their affiliated organizations, or those of the publisher, the editors and the reviewers. Any product that may be evaluated in this article, or claim that may be made by its manufacturer, is not guaranteed or endorsed by the publisher.

## Footnotes

^1^ https://pubmlst.org/species-id

^2^ https://www.ezbiocloud.net/

^3^ https://pubmlst.org/organisms

^4^ https://cge.cbs.dtu.dk/services/SCCmecFinder/

^5^ https://www.sccmec.org/index.php/en/
