# Supplementary material for: Molecular epidemiology and antibiotic resistance of staphylococci other than Staphylococcus aureus in children in Cape Town, South Africa
Source: Front Microbiol. 2023 Aug 2;14:1239666. doi: 10.3389/fmicb.2023.1239666 (PMC10437061; doi:10.3389/fmicb.2023.1239666)
Supplement: Supplementary file 1 [file Table_1.DOCX]

**STANDARD OPERATING PROCEDURE (SOP) - Study Specific stool collection aspects for TB CHAMP.**

**Objectives**

This SOP describes the procedure for collection of stool specimens for the purpose of bacterial culture and molecular microbiome studies, in the context of the TB-CHAMP study.

**Procedures**

***Equipment***

**Table 1: Equipment required**

| **STOOL COLLECTION** | |
| --- | --- |
| 1 | Sample container. Faecal Container (screwcap) With Spoon (25 ml) (LASEC product number: PLPS109047) |
| 2 | Leak-proof bag |
| 3 | Opaque carrier to protect dignity |
| 4 | Cling-wrap (optional) |
| 5 | Potty/Bed-pan (optional) |
| **TRANSPORT** | |
| 6 | Labels and indelible marker pen |
| 7 | Transport coolbox |
| 8 | Icebrick |
| 9 | Stool requisition form |
| **SAMPLE PROCESSING** | |
| 10 | Cotton Swabs |
| 11 | Cary Blair Medium |
| 12 | DNA buffer (if used) or -80 freezer |

***Stool collection***

1. **Procedure for clinic collection**
   1. Study nurse must collect stool as per DTTC stool collection SOP (PC011 Stool Collection Version 1.0)
   2. Make sure no urine, water, soil or other material gets in the container. An attempt should be made to collect a specimen that consists primarily of stool when only a mixture of stool and urine is available. The stool should be mixed with the spoon at least five times to generate a homogeneous mixture. At least 6 scoops of stool needs to be added into the container. If the stool is loose, collect at least 3 scoops of watery stool (only if it is free from urine as stated above) and 3 of more solid stool.
   3. Ensure that the specimen containers are sealed well and disinfect the outside with 70% alcohol if necessary. The container should be placed in a sealable plastic bag. Label the collection container with the Subject Event barcode.
   4. The clinician / study nurse should complete the stool requisition form (DTTC TB-CHAMP Stool Requisition Form V1.2
   5. Keep specimen in the fridge (2-8°C) at the site and transport to Tygerberg Hospital in coolboxes on ice on the same day as collection, or as soon as possible. Avoid freezing the sample as subsequent thawing during transport may cause DNA degradation.
2. **Procedure for home collection**
   1. Clear and simple verbal instructions regarding the method of stool collection should be given to the caregiver by the study nurse or counsellor, along with the instruction leaflet (TB-CHAMP_Parent Instruction Leaflet_V1.0).
   2. The caregiver should be given a stool container, leak-proof bag and opaque plastic box
      1. In addition, if the caregiver has a freezer at home, a coolbox and icebrick should be provided. The icebrick must be kept in the freezer until required (see 2.4)
   3. Encourage the caregiver to put the stool sample in the collection container provided by the study team, and to note the time and date of stool collection - preferably taken as soon as possible before the clinic visit (night before, or ideally the morning of the clinic visit).
   4. Once collected, the sample should be kept on the icebrick in a coolbox (if provided), or in the coolest place in the home until delivered to clinic.
      1. The study team must inform the caregivers that stool should not be collected on a Friday afternoon as the sample will only reach DTTC on the Monday.
   5. For baseline (BL) stool should be taken at screening visit, and if not obtained a sample collection kit will be provided for home collection. The kit and sample will then be returned at BL visit.
      1. In cases where screening and BL occur on the same day, the stool must be collected before treatment/placebo is issued. If this is not possible, the participant must be excluded from future analyses regarding stool.
      2. For 8-, 16- , 24- and 48-week follow-up samples, if participants are unable to deliver a stool sample at the clinic, a sample collection kit will be provided to them for home collection. A driver will pick-up the samples from the home.
      3. The study team should follow-up with the caregivers everyday after the visit until they can confirm that a sample was taken and collected.

***Transport***

1. When a driver collects a stool sample (from clinic OR participant home), he / she should
   1. Have a cooler box and frozen ice brick ready for transporting stool samples
   2. Wear gloves, ensure that the collection container is sealed and disinfect the outside with 70% alcohol if necessary
   3. Fill out the stool requisition form (if not completed by clinician / study nurse (1.4) and ensure the Subject Event barcode is pasted on the collection container.
2. Preparation of specimen and transport to the Laboratory
   1. The specimen must be transported to the Division of Medical Microbiology (Room 561, 9^th^ Floor, Green Lane East, Tygerberg Hospital) as soon as possible in a cooler box with an ice pack to keep it at 2-8°C during transport.
   2. If delivery to TBH is unsuccessful, the sample should be brought to the DTTC (Room 0065, K-floor, Stellenbosch University Medical School) and stored at -80°C. This should also be noted in the comment section of the requisition form.
   3. A WhatsApp group called “TB-CHAMP Stool” will be created to enable communication between the Medical Microbiology laboratory team members, at least one member from the DTTC laboratory and the drivers.

***NHLS Section Only: Sample processing***

1. Upon arrival at the Medical Microbiology laboratory, the stool requisition form will be signed by the recipient, noting the time and date of arrival.
2. The sample will be split into two as soon as possible
   1. A swab or spoonful of stool is to be transferred to Cary Blair medium for culture work.
   2. A spoonful of stool is to be transferred to DNA stabilizing buffer (if used)
   3. The remainder of the sample will be frozen and stored at -80°C
   4. A separate sample barcode will be created for each sample split as outlined above and placed on the requisition form.
   5. The QC of the requisition form will be finalised by the Medical Microbiology TB-CHAMP personnel.
